# English Stop-Smoking Services: One-Year Outcomes

**DOI:** 10.3390/ijerph13121175

**Published:** 2016-11-24

**Authors:** Linda Bauld, Rosemary Hiscock, Fiona Dobbie, Paul Aveyard, Tim Coleman, Jo Leonardi-Bee, Hayden McRobbie, Andy McEwen

**Affiliations:** 1 Institute for Social Marketing, University of Stirling, Stirling FK9 4LA, UK; fiona.dobbie@stir.ac.uk; 2 UK Centre for Tobacco and Alcohol Studies; r.hiscock@bath.ac.uk (R.H.); paul.aveyard@phc.ox.ac.uk (P.A.); tim.coleman@nottingham.ac.uk (T.C.); jo.leonardi-bee@nottingham.ac.uk (J.L.-B.); h.j.mcrobbie@qmul.ac.uk (H.M.); andy.mcewen@ncsct.co.uk (A.M.); 3 Department for Health, University of Bath, Bath BA2 7AY, UK; 4 Nuffield Department of Primary Care Health Sciences, University of Oxford, Radcliffe Observatory Quarter, Woodstock Road, Oxford OX2 6GG, UK; 5 Division of Primary Care, University of Nottingham, Nottingham NG7 2RD, UK; 6 Division of Epidemiology and Public Health, University of Nottingham, City Hospital Campus, Hucknall Road, Nottingham NG5 1PB, UK; 7 Wolfson Institute of Preventive Medicine, Queen Mary University of London, London EC1M 6BQ, UK; 8 National Centre for Smoking Cessation and Training (NCSCT), 1 Great Western Industrial Centre, Dorchester DT1 1RD, UK

**Keywords:** smoking cessation, stop-smoking services, smoking cessation services, behavioural support, pharmacotherapy

## Abstract

The UK is a global leader in stop-smoking support—providing free behavioral support and cessation medication via stop smoking services (SSS) without charge to smokers. This study aimed to explore the client and service characteristics associated with abstinence 52 weeks after quitting. A prospective cohort study of 3057 SSS clients in nine different areas of England who began their quit attempt between March 2012 and March 2013 was conducted. Important determinants of long-term quitting were assessed through quit rates and multivariable logistic regression. Our results showed that the overall weighted carbon monoxide validated quit rate for clients at 52 weeks was 7.7% (95% confidence interval (CI) 6.6–9.0). The clients of advisors, whose main role was providing stop-smoking support, were more likely to quit long-term than advisors who had a generalist role in pharmacies or general practices (odds ratio (OR) 2.3 (95% CI 1.2–4.6)). Clients were more likely to achieve abstinence through group support than one-to-one support (OR 3.4 (95% CI 1.7–6.7)). Overall, one in thirteen people who set a quit date with the National Health Service (NHS) Stop-Smoking Service maintain abstinence for a year. Improving abstinence is likely to require a greater emphasis on providing specialist smoking cessation support. Results from this study suggest that over 18,000 premature deaths were prevented through longer-term smoking cessation achieved by smokers who accessed SSS in England from March 2012 to April 2013, but outcomes varied by client characteristic and the type of support provided.

## 1. Introduction

The UK stop-smoking services (SSS) have aimed to reduce smoking-related deaths, particularly from cancer and coronary heart disease, which predominantly occur among disadvantaged groups since 1999 [1,2]. SSS provide behavioural support delivered by trained practitioners and pharmacotherapy. SSS clients are routinely followed up four weeks after they have set a quit date. Self-report and carbon monoxide (CO) tests are undertaken. Previous studies have shown that the services reach large numbers, achieve a substantial impact and are cost effective [3,4,5]. Recent routine data from the SSS in England show that 35% of service users achieve Russell standard carbon monoxide abstinence four weeks after their quit date (the date at which they agree with the services that they will stop smoking), which equates to 72,111 four-week quitters per year [6]. Measuring longer-term abstinence is resource intensive due to growing lost to follow-up rates and thus the English services do not have the capacity for long-term follow-up [7].

The English SSS are based on a body of evidence surrounding optimal smoking cessation techniques, with ongoing audit and evaluation [8,9,10]; two previous studies collected long-term (52-week) follow-up data from UK SSS. The first of these was conducted in 2002 in Nottingham and North Cumbria. Fifteen percent of 2069 clients had been abstinent for a year and were CO validated after one year [7]. In 2007 data from 1374 pharmacy services and 411 closed group clients in Glasgow were collected. The CO validated quit rate at 12 months was 2.8% for the pharmacy service and 6.3% for the closed groups. Nevertheless, both types of service were found to be cost effective [3].

Other than England, the UK nations attempt to collect 52-week self-report follow-up data. During the financial year April 2012 to March 2013, the Welsh services, where clients mostly attend closed groups, had the highest quit rate (32%) but the lowest reach (6299 clients) [11] whereas the Scottish services, which are 75% pharmacy led, had the lowest quit rate (6%) but a reach of 116,198 quit attempts [12]. The Northern Irish services have an intermediate reach (32,714 quit attempts) and a quit rate of 17% (for 2011/2012, latest data available) [13]. For comparison, 724,247 quit attempts were made with the English services 2012/2013 [14] with no national data on 52-week outcome.

In 2011, the English Department of Health commissioned a new study to examine longer-term abstinence among clients of the English SSS: ‘Evaluating longer-term outcomes of the National Health Service (NHS) Stop Smoking Services’ (ELONS). It was commissioned for two key reasons. First, was to fill an evidence gap (there has been no comprehensive, independent evaluation of English stop-smoking services’ long-term outcomes for a decade). Second, the configuration of the stop-smoking services has changed markedly (group support has declined in favour of one-to-one support [6,14] and a growing proportion of SSS clients received support from non-specialist practitioners. These are people whose main job is providing general NHS care but who have had some training to also provide SSS behavioural support. Usually non-specialist advisors are general practice (GP) nurses, healthcare assistants, pharmacists, and pharmacy assistants [15,16,17,18]. It is important to assess whether these changes could influence longer-term outcomes.

## 2. Materials and Methods

Ethical approval was obtained in June 2011 from NHS Lothian (South East Scotland Research Ethics Committee 2003). The research took place in nine areas of England: Bristol; County Durham and Darlington; Hull and East Riding; Leicestershire County and Rutland; North and North East Lincolnshire; Northamptonshire; Oldham; Rotherham; and South East Essex. Services in these areas were asked to participate because they encompassed a range of behavioural support types, short-term success rates, urban and rural locations [19] and they used QuitManager (North 51, Nottingham, UK) [20] for routine monitoring data management (so electronic data on participants’ characteristics in a standard format was available to the research team). General practices (NHS provided centres of family medicine) and pharmacies (private companies which dispense medicines) with staff who were trained SSS advisors within these areas were also asked to participate in the study.

SSS clients who set a quit date, were not pregnant and aged 16 years or over, were recruited to the study between March 2012 and March 2013; the majority (77%) between July and November. Clients attended a number of behavioural support sessions with a trained advisor and pharmacotherapy was available. Additional data were collected from participating clients on sociodemographic characteristics, health and wellbeing, smoking behaviour, pharmacotherapy and behavioural support type. These data were collected either by advisors (seven sites) and entered directly into QuitManager or, where sites did not have the capacity for advisors to collect additional data, by a Primary Care Research Network (PCRN) researcher on paper which was then manually entered by the research team.

Self-reported quitters at four weeks were contacted by TNS-BMRB (a social research company, Westgate, London, UK) 52 weeks after they had set a quit date and asked to complete a short telephone questionnaire about their smoking status. Those that reported not smoking in the last seven days were asked if they would participate in a CO monitoring test at their home. At least three attempts were made to contact each client, at different times of day, before they were classified as lost to follow-up.

The 52-week follow-up and descriptive information were combined in an anonymised database for analysis. Sample representativeness of the sample and weight creation required a comparison of the ELONS sample with a dataset including every client, not just those who participated in ELONS, from the nine participating services. These clients had a quit date set during the recruitment period (March 2012 to March 2013). The dataset was provided by North51 with a limited selection of client characteristics, service characteristics and four-week quit data that is routinely collected.

We also had access to the datasets of two aforementioned previous long-term evaluations of the UK Stop Smoking Services: the Nottingham/North Cumbria study in 2002 [7] and Glasgow in 2007 [3]. Quit rates and follow-up rates from these studies were compared with the ELONS study.

### 2.1. Sample

Data on 3075 quit attempts were collected, six clients (0.2%) were excluded due to pregnancy and 12 (0.4%) were excluded because details about the client’s behavioural support advisor were not recorded in the database. The final study sample (*n* = 3057) represents 5% of all those recorded as using the English SSS services during the recruitment period (March 2012 to March 2013). Of these 3057 cases, 1729 (56.6%) self-reported four-week abstinence and were eligible for 52-week follow-up.

### 2.2. Measures

Characteristics studied include design variables, demographic variables, socioeconomic status (SES); health and wellbeing, dependence on tobacco, support, determination to quit; and pharmacotherapy.

#### 2.2.1. Design Variables

Design variables needed to be included in all regression models because of differences between the study sample and the client base of the services: we deliberately oversampled clients who received behavioural support in a group setting in order to have sufficient cases for analysis. General practices and pharmacies proved difficult to recruit to the study, some services recruited higher proportions of their clients than others and services recruited over different time periods. We included six different forms of behavioural support, four forms were run by specialist advisors: open groups, where clients can join the group at any time; closed groups, where clients start the course together and new clients are not added to the group after the first session; one-to-one sessions, where clients meet individually with an advisor; and drop-ins where clients can attend without previously making an appointment. The fifth form of behavioural support is provided in general practices and the advisors are general practice employees who do smoking cessation work as one of many tasks. The sixth form of behavioural support took place in pharmacies and the advisors were pharmacy staff who again provide smoking cessation advice alongside other tasks. The locations were nine areas of England. Attendance at SSS varies with time of year, thus we separated clients who attended after Christmas (January and February) which tend to be busy months with high quit rates from those who attended in the quiet months in the Summer (July and August) and who attended after the summer break (September and October) from those who attended in other months.

#### 2.2.2. Socio-Demographic Variables

We recorded age, gender and ethnicity to measure demography. To measure socioeconomic status, we created a variable which counted the number of the following indicators of disadvantage: (1) routine or manual occupation or unemployed or permanently sick; (2) basic (general certificate of secondary education, GCSE) or no education; (3) social or private renting; (4) eligible for free prescriptions; (5) single parent. We contrasted clients with 0 to 1 indicators of disadvantage with clients with three to five indicators of disadvantage. We have used similar methodology for deriving an overall SES measure from a plethora of indicators in previous evaluations of SSS [7,21,22].

#### 2.2.3. Health and Wellbeing

Low levels of mental wellbeing have been associated with low cessation rates [23]. We used the WHO-Five wellbeing scale [24] to measure health and wellbeing. This is a validated, internationally used, five item, Likert type scale which measures hedonic (emotional) and eudemonic (meaning) aspects of subjective wellbeing [25]. Responses to the five items are summed to produce a wellbeing score. A higher score indicates higher wellbeing.

#### 2.2.4. Dependence on Tobacco, Support and Determination to Quit

Measures of smoking related characteristics included dependence. Clients who smoked within five minutes of waking or who smoked more than 30 cigarettes a day were classified as dependent.

We measured support through two variables: whether clients had support from a spouse or partner for their quit attempt and whether half or more of their family and friends did not smoke. Clients who said that they were extremely or very determined to quit were classified as determined to quit.

#### 2.2.5. Medication

The medication given varied between locations. As it was necessary to include location in the model for sampling reasons, we were only able to include whether clients took varenicline, a particularly effective medication [26,27].

#### 2.2.6. Potential Confounders Excluded

The following characteristics were not used in the analysis due to a non-significant association with quitting smoking and/or multicollinearity in preliminary testing: marital status, medical conditions, some medication types (single nicotine replacement therapy (NRT), multiple NRT, bupropion (services medication regimens differed widely so there was collinearity with medication)), how introduced to the services, previous quit attempt, advisor type, and intervention setting.

#### 2.2.7. Outcomes

The main outcome, consistent with the Russell Standard [28], was CO validated quitting at 52 weeks, as a proportion of everyone who tried to quit. We took an intention-to-treat approach, thus clients who were lost to follow-up were counted as smoking. Thus, we were able to include all clients present at baseline in the analysis of long-term quitting.

The Russell Standard allows clients to still be counted as continuously abstinent (or quit at 52 weeks) if they have smoked up to five cigarettes during the abstinence period. For completeness, we also calculated various alternatives: a stricter quit rate which does not allow any lapses (known as ‘not a puff’ [29]) and a more lenient quit rate (known as ‘point prevalence’ [30]) which only asks whether the client has smoked within the past seven days.

### 2.3. Procedure

Firstly, CO validated and self-report quit rates and follow-up statistics were calculated and tabulated with the Nottingham/North Cumbria 2002 data and the Glasgow 2007 data.

Secondly, for each characteristic, raw and weighted 52-week CO validated quit rates were calculated. As a result of the low participation rate, the sample was rim-weighted to the characteristics of the population of clients who passed through the nine participating services between March 2012 and March 2013. The variables used for weighting were behavioural support type, age, gender and SES (National Statistics Socio-Economic Classification (NS-SEC) [31]).

Quit rates were then calculated using weighted data and accounting for clustering by location. SPSS (v21) (IBM, New York, NY, USA) was used to calculate the raw quit rates and Stata (v13) (StataCorp LP, College Station, TX, USA) was used to calculate corrected quit rates and 95% confidence intervals (CI).

Thirdly, the relationships between CO validated quitting, personal and stop-smoking service characteristics were investigated with logistic regression analysis using SPSS (v21). Initially, multilevel models were created with the client at level 1 and the advisor at level 2. However, due to lack of variance at level 2 (between advisors) the multilevel models were redundant; therefore, standard logistic regression models were used, with design variables and sociodemographic variables (with the exceptions of ethnicity and wellbeing) included as a priori variables. For the other predictors (including ethnicity and wellbeing) a backwards stepwise elimination procedure was used. Multicollinearity was tested by entering each predictor into the model alone and comparing the standard error with the final model. An increase greater than 5% was taken as indicating a concerning level of multicollinearity [32]. None of the variables in the final model had indications of multicollinearity. Model robustness was tested by adding variables that had been non-significant in preliminary modelling of four-week outcomes: how introduced to service, medication type (which was substituted for the medication variable), health and wellbeing alternatives (substituted for the wellbeing WHO-Five score) and marital status. None of these reached significance.

## 3. Results

### 3.1. Smoking Status

Less than one quit attempt in 10 (9.3%) was CO validated as achieving long-term abstinence (consistent with the Russell Standard), rising to 14.7% with the inclusion of self-reported cases without CO validation (Table 1). There were 41.7% who were not long-term abstinent (which included 0.8% where the CO validation was challenged by a CO ≥10 parts per million test), with a further 43.6% lost to follow-up. Short-term (four-week) abstinence was achieved by 57% of clients. Follow-up was achieved for 34% of baseline clients. Ten percent were given CO validation tests. The CO validated point prevalence quit rate was 18.3% and the quit rate including only clients who stated they had not had any cigarettes (“not a puff”) was 12.8%.

The long-term abstinence rate was intermediate between the Nottingham/North Cumbria evaluation (14.6%) and the Glasgow evaluation (3.6%). Follow-up losses were smaller in the former and larger in the latter study.

### 3.2. Fifty-Two-Week Quit Rates and Multivariable Odds Ratios for Predicting Quitting for Various Client Groups

#### 3.2.1. Sample Design Characteristics

The weighted quit rate reduced to 7.7% (95% CI 6.6–9.0) from a raw quit rate of 9.3% (Table 2). For analysis purposes, open and closed groups and non-specialist general practitioner and pharmacy services were combined. Over twice as many clients quit who received specialist group and specialist one-to-one behavioural support than clients who received general practitioner and pharmacy based behavioural support (quit rates were 12.1% (95% CI 10.5–13.8) 10.2% (95% CI 7.6–13.7) and 5.1% (95% CI 2.8–9.3) respectively). After taking into account client and service characteristics, the odds of quitting were three times higher among group clients and two times higher among clients who received specialist one-to-one support compared with general practitioner and pharmacy based support (adjusted odds ratios (ORs) 3.4 (95% CI 1.7–6.7) and 2.3 (95% CI 1.2–4.6) respectively).

There were no significant differences by location. The New Year period was the most successful time of year for quitting smoking long-term (quit rate 13.1% (95% CI 5.1–29.6)) and this was significantly more successful than the summer holiday period (quit rate 6.3% (95% CI 4.4–8.9) adjusted OR 1.7 (95% CI 1.0–2.9)).

#### 3.2.2. Sociodemographic Characteristics

Older clients were more likely to quit for each year of age that the increased odds ratio of quitting was 1.011 (95% CI 1.002–1.020). Affluent clients were more likely to quit than socioeconomically disadvantaged clients (quit rates 10.3% (95% CI 8.4–12.7) and 6.2% (95% CI 5.0–7.7) respectively, (adjusted OR 1.4 (95% CI 1.1–1.9)). The mean wellbeing score of quitters was 59.3 out of 100 (95% CI 56.5–62.1) which was significantly higher than the score for non-quitters which was 52.7 out of 100 (95% CI 51.4–53.9) for non-quitters (adjusted OR 1.007 (95% CI 1.0003–1.013)). Gender differences did not reach significance in multivariable modelling and ethnicity was eliminated by the backwards stepwise procedure.

#### 3.2.3. Smoking and Quit Attempt Characteristics

Quit rates were higher among clients who started a quit attempt taking varenicline (quit rate 10.0% (95% CI 7.2–13.8) compared with 6.2% (95% CI 4.9–7.7) adjusted OR 1.7 (95% CI 1.3–2.3)), clients who were less dependent on tobacco (quit rate 9.8% (95% CI 7.7–12.4) compared with 4.9% (95% CI 2.9–8.2), adjusted OR 1.5 (95% CI 1.1–1.9)), clients whose quit attempt was supported by a spouse or partner (quit rate 9.2% (95% CI 7.4–11.3) compared with 6.2% (95% CI 4.5–8.5), adjusted OR 1.4 (95% CI 1.0–1.8)) and clients whose social network contained fewer smokers (9.1% (95% CI 7.5–10.9) compared with 3.4% (95% CI 2.6–4.4)) (adjusted OR 2.0 (95% CI 1.4–2.9)). Determination to quit was eliminated by the backwards stepwise procedure.

## 4. Discussion

This study provides new evidence that government funded stop-smoking programmes do enable smokers to quit long-term. The long-term quit rate of smokers who do not participate in a programme is approximately 3% [34] which is lower than the overall quit rate here (8%) and particularly lower than those of the specialist one-to-one and group services (10% (95% CI 8%–11%) and 12% (95% CI 11%–14%) respectively). Consistent with a growing number of studies [3,16,35,36], the most effective behavioural support was provided by specialist advisors. This implies that service commissioners need to continue to invest in this type of provision to deliver better outcomes for smokers trying to quit.

Why might the specialist provision be superior? There is emerging data that general practices and pharmacies may lack the resources to monitor clients sufficiently and develop their services [16,37,38]. Additional financial incentives may also be needed to encourage busy staff with multiple roles to prioritise smoking cessation above other tasks [38]. General practice staff are under increasing pressure due to declining funding and a growing population in the UK [39]. Thus, time pressure may be too great to ensure quality behavioural support, even with extra resources in place [37]. Thus, it may be preferable for GP practice and pharmacy employees to offer only brief support to use stop-smoking medication and refer to specialist services if the smoker would like behavioural support to stop. Nevertheless, this study did not include a cost effectiveness element and only 12% of the sample were GP practice and pharmacy clients. A previous study of pharmacy services in Glasgow did conclude that they were cost effective [3]. Thus, further research is needed.

Overall, results were consistent with some longer-term routine monitoring data from services in other parts of the UK outside England. For example, a self-report quit rate of 15% was similar to Northern Ireland and lower than Wales where groups predominate, and higher than Scotland where behavioural support provided in pharmacies is the dominant service model.

The longer-term CO validated quit rate in this study was somewhat lower than the previous evaluation of the English SSS conducted a decade before our research. The main difference was in loss to follow-up. This may not only reflect an increase in non-quitters. Since 2002, many people have moved from landline telephones to mobile telephones [40]. People tend to change their mobile phone number much more frequently than a landline number [41], thus participant contact phone numbers are more likely to change between baseline and follow-up. Additionally, refusal rates are higher [42]. For the telephone 52-week follow-up, 8% had moved, died or were no longer capable of being interviewed, 5% refused and 24% did not answer or were unavailable. This may have been exacerbated by a market research company undertaking the ELONS study long-term follow-up whereas initial contact for the follow-up in the previous evaluation was conducted by SSS staff who already had a rapport with clients.

### 4.1. Limitations

This was an observational study, thus there may have been unmeasured confounders and we need to be cautious about attributing causality. Nevertheless, it is important to study smoking cessation in real-world settings. Due to confounding with location, we were unable to include NRT in the analysis, so it is possible that some of the variation between specialist and GP and pharmacy based services could have been due to variation in use of NRT. Preliminary analysis suggested that single NRT and combination NRT were not strongly associated with high quit rates in this study, when compared with varenicline [33]. However, given that varenicline was included, this may have captured the variance that some SSS were using perhaps less NRT in favour of more varenicline. There was lower recruitment to the prospective study than we anticipated. Since our last study, the required length and complexity of the consent process grew and this seems to have deterred both smoking cessation advisors and clients from participating. Additionally, the services were undergoing a period of change which included redundancies and reduced working conditions which may have reduced motivation among advisors. Weighting quit rates and multivariable analysis helped overcome a lower than expected response rate.

### 4.2. Impact

ELONS results can also be used to estimate that 36,249 smoking-related premature deaths were prevented as a result of English stop-smoking services in the financial year 2012–2013, given that 724,247 quit dates were set, 8% of ELONS participants were abstinent at one year, 35% of those were abstinent for one-year relapse [43] and half of regular smokers die prematurely from smoking related diseases.

## 5. Conclusions

The English stop-smoking services are still helping large numbers of smokers to quit long-term. The specialist services, particularly group services, appear to be most successful and offer smokers the best chance of success in their quit attempt. Continued delivery of stop-smoking services forms an important part of comprehensive tobacco control.

## Figures and Tables

**Table 1:** Quit rates and follow-up rates.

	Nottingham/North Cumbria (*n* = 2069) 2002 N (%)	Glasgow (*n* = 1785) 2007 N (%)	ELONS ^1^ (*n* = 3057) 2012/2013 N (%)
*52 weeks follow-up (Russell Standard):*			
1 CO validated quit (0–5 cigarettes since quit date)	303 (14.6)	64 (3.6)	285 (9.3)
2 Self report not CO validated	65 (3.1)	63 (3.5)	165 (5.4)
3 Self report refuted by CO validation	8 (0.4)	1 (0.1)	25 (0.8)
4 Non-quitters at 52 weeks	525 (25.4)	264 (14.8)	578 (18.8)
5 Non-quitters at 4 weeks	392 (18.9)	259 (14.5)	681 (22.1)
6 Lost to follow-up at 52 weeks	367 (17.7)	179 (10.0)	689 (22.4)
7 Lost to follow-up at 4 weeks	409 (19.8)	955 (53.5)	653 (21.2)
*Total 52-week self-report (excluding refuted by CO test)*	368 (17.7)	127 (7.1)	450 (14.7)
*Alternative self-report quit rates at 52 weeks* ^2^			
Point prevalence ^3^	NA	131 (7.3)	558 (18.3)
Continuous abstinence (not a puff)	377 (18.2)	108 (6.1)	390 (12.8)
*Alternative CO validated quit rates at 52 weeks*			
Point prevalence ^3^	NA	NA	348 (11.4)
Continuous abstinence (not a puff)	303 (14.6)	62 (3.5)	260 (8.5)
*Total eligible for follow-up at 52 weeks (all 4 weeks self-report)*	1272 (61.5)	568 (31.8)	1735 (56.7)
Total *successfully* followed up at 52 weeks	901 (43.5)	392 (22.0)	1051 (34.4)
Total eligible for CO validation at Russell standard	376 (18.2)	128 (7.2)	475 (15.5)
Total given CO test	311 (15.0)	65 (3.6)	310 (10.1)
CO validated quit rate of those successfully followed up	34.5%	16.1%	27.1%

^1^ Unweighted; ^2^ These include all self-report (includes refutes); ^3^ Whether not smoked within: ELONS the last 7 days, Glasgow last 2 weeks. CO: carbon monoxide; NA: not applicable. This table is reproduced from the project report [33].

**Table 2:** ELONS 52-week raw and weighted CO validated quit rates at 52 weeks (percents and 95% confidence interval (CI)), weighted mean age and wellbeing (and weighted 95% CI) and adjusted odds ratios from multivariable logistic regression model of CO validated quitting.

	N	%	Quit Rate	Weighted Quit Rate (95% CI)	Multivariable Logistic Regression OR (95%CI)
Total	3057	100	9.3	7.7 (6.6–9.0)	
*Behavioural support (truncated)*					
group specialist	652	21.3	11.8	12.1 (10.5–13.8)	3.4 (1.7–6.7)
drop in specialist	887	29.0	7.9	7.6 (5.1–11.0)	1.7 (0.9–3.5)
one-to-one specialist	1131	37.0	10.4	10.2 (7.6–13.7)	2.3 (1.2–4.6)
general practice or pharmacy service	366	12.0	4.9	5.1 (2.8–9.3)	1.0
other or unknown	21	0.7	9.5	Not available	2.3 (0.5–11.6)
*Location*					
1	273	8.9	7.7	6.6 (3.9–11.1)	1.0
2	741	24.2	10.4	8.1 (6.4–10.3)	1.1 (0.6–2.1)
3	88	2.9	11.4	11.0 (5.7–20.2)	1.3 (0.5–3.3)
4	396	13.0	10.9	9.3 (6.3–13.5)	1.2 (0.6–2.3)
5	74	2.4	5.4	4.2 (1.3–12.6)	0.7 (0.2–2.4)
6	690	22.6	10.0	7.8 (5.0–11.9)	0.7 (0.4–1.2)
7	146	4.8	6.2	6.4 (3.3–12.2)	0.9 (0.3–2.3)
8	555	18.2	8.3	8.0 (5.9–10.7)	1.1 (0.5–2.1)
9	94	3.1	6.4	11.9 (4.2–29.5)	0.9 (0.3–2.6)
*Recruitment date*					
other months	767	25.1	9.5	7.0 (5.2–9.4)	1.2 (0.8–1.7)
Summer—July, Aug	970	31.7	8.1	6.3 (4.4–8.9)	1.0
back to school—Sept, Oct	1128	36.9	9.7	8.7 (6.4–11.7)	1.2 (0.9–1.6)
New Year—Jan, Feb	192	6.3	12.5	13.1 (5.1–29.6)	1.7 (1.0–2.9)
*Age* *(weighted mean (years))*			*not quit 43.3 (42.5–44.1)* *quit 46.8 (44.4–49.2)*		1.011 (1.002–1.020)
*Gender*					
female	1710	55.9	8.5	7.2 (6.0–8.5)	1.0
male	1347	44.1	10.4	8.4 (6.8–10.2)	1.2 (0.9–1.5)
*Ethnicity*					
white British	2866	93.8	9.4	7.4 (6.1–9.0)	not entered
other white	69	2.3	10.1	11.5 (4.5–26.0)	
Asian (including mixed white and Asian)	64	2.1	3.1	3.6 (1.3–9.5)	
Other and unknown	58	1.9	12.1	21.6 (7.0–50.1)	
*SES*					
0–1 indicators of low ses	1123	36.7	13.0	10.3 (8.4–12.7)	1.4 (1.1–1.9)
2–5 indicators of low ses	1934	63.3	7.2	6.2 (5.0–7.7)	1.0
*WHO_5 Wellbeing (weighted mean)*			*not quit 52.7 (51.4–53.9)* *quit 59.3 (56.5–62.1)*		1.007 (1.0003–1.013)
*Medication in week 1*					
varenicline not recorded	1661	54.3	6.7	6.2 (4.9–7.7)	1.0
took varenicline	1396	45.7	12.4	10.0 (7.2–13.8)	1.7 (1.3–2.3)
*Dependence*					
other	1681	55.0	11.4	9.8 (7.7–12.4)	1.5 (1.1–1.9)
highly dependent	1376	45.0	6.8	4.9 (2.9–8.2)	1.0
*Determination to quit*					not entered
other	328	10.7	7.6	5.9 (4.3–8.0)	
very/extremely determined	2729	89.3	9.5	8.0 (6.7–9.5)	
*Support from spouse partner*					
other	1507	49.3	7.3	6.2 (4.5–8.5)	1.0
support from spouse/partner	1550	50.7	11.3	9.2 (7.4–11.3)	1.4 (1.0–1.8)
*Friends and family*					
other	771	25.2	4.7	3.4 (2.6–4.4)	1.0
half or fewer smoke	2286	74.8	10.9	9.1 (7.5–10.9)	2.0 (1.4–2.9)

SES: socioeconomic status.
